# Tetanus Toxoid Vaccination Coverage and Associated Factors among Childbearing Women in Ethiopia: A Systematic Review and Meta-Analysis

**DOI:** 10.1155/2021/5529315

**Published:** 2021-11-08

**Authors:** Jemberu Nigussie, Bekahegn Girma, Alemayehu Molla, Moges Mareg

**Affiliations:** ^1^Department of Nursing, College of Health Sciences and Medicine, Dilla University, Dilla, Ethiopia; ^2^Department of Psychiatry, College of Health Science and Medicine, Dilla University, Dilla, Ethiopia; ^3^Department of Reproductive Health, School of Public Health, College of Health Science and Medicine, Dilla University, Dilla, Ethiopia

## Abstract

**Background:**

Tetanus is a bacterial disease caused by the Clostridium tetani, which is a highly fatal, noncommunicable, and toxin-mediated disease. Globally, maternal and neonatal tetanus is a public health problem due to low maternal tetanus toxoid immunization. Ethiopia has the highest neonatal mortality and morbidity related to tetanus due to low tetanus toxoid immunization and the high number of home deliveries. The main objective of this systematic review and meta-analysis was to estimate the pooled coverage of at least two doses of tetanus toxoid immunization, and the pooled effect sizes of associated factors in Ethiopia.

**Methods:**

Primary studies for this review were searched from the PubMed/MEDLINE online, ScienceDirect, Hinari, Google, and Google Scholar databases. Primary articles published from 2010 up to August 30, 2020, were included in this meta-analysis. Data were extracted in Microsoft Excel format and exported to STATA Version 14.0. A random-effects meta-analysis model was used to estimate the pooled coverage of two or more tetanus toxoid immunizations and its associated factors. Heterogeneity was evaluated by the *I*^2^ test. Egger's weighted regression test was used to assess publication bias.

**Results:**

We retrieved 212 records; of these, 199 articles were excluded for reasons. Finally, 14 studies were included in this meta-analysis. The pooled estimate of receiving at least two doses of tetanus toxoid immunization coverage in Ethiopia was 52.2% (95% CI: 42.47-61.93, *I*^2^ = 98.4%). Antenatal care (OR = 7.8 (95% CI: 3.2, 19.2), *I*^2^ = 96.3%), media exposure (OR = 8.3 (95% CI: 2.1, 33.3), *I*^2^ = 98.1%), distance from the health facility (OR = 2.64 (95% CI: 1.1, 6.6), *I*^2^ = 94.1%), educational status of women (OR = 4.7 (95% CI: 2.07, 9.56), *I*^2^ = 94.2%), and educational status of husbands (OR = 2.995 (95% CI: 1.194, 7.512), *I*^2^ = 92.5%) were factors significantly associated with receiving at least two doses of tetanus toxoid immunization coverage in Ethiopia.

**Conclusions:**

The coverage of tetanus toxoid immunization among childbearing women was low in Ethiopia. Strengthening maternal health service utilization (antinatal care and institutional delivery) to the nearest health facility even in health posts and empowering education for both women and their husbands is recommended to increase tetanus toxoid immunization coverage in Ethiopia.

## 1. Introduction

Tetanus is a bacterial disease caused by the Clostridium tetani, which is a highly fatal, noncommunicable, and toxin-mediated disease [1, 2]. Due to unhygienic deliveries and low tetanus toxoid (TT) immunization, women and their newborns are high risk of acquiring tetanus [3–7]. Globally, maternal and neonatal tetanus (MNT) is a public health problem due to low maternal TT immunization [8]. Annually, 3.3 million neonatal deaths occur, of which neonatal tetanus shares a high proportion (more than half million neonatal deaths), particularly in developing countries where home delivery is common [6, 9]. Every year, 15,000–30, 000 maternal deaths occur due to tetanus related to the delivery process [10]. The World Health Organization (WHO) report showed that 90% of maternal and neonatal tetanus occurred in South East Asian (SEA) and Sub-Saharan African (SSA) countries, and almost all cases end with death [11].

The main strategies for the prevention of maternal and neonatal tetanus (MNT) at birth were vaccination of the mothers with protective dose of tetanus toxoid (TT) immunization and clean delivery [3, 12, 13]. Nearly 94% of neonatal mortality related to tetanus can be reduced by immunization of women of childbearing age with at least two doses of tetanus toxoid immunization (TT^2+^) [14]. The WHO recommends 5 consecutive doses of tetanus toxoid (TT) vaccination for women of childbearing age (CBAW) per schedule for lifelong protection against tetanus [1].

In many countries, at least two doses of TT vaccination can be administered to pregnant women during antenatal care visits [15]. TT^2+^ immunization coverage among pregnant mothers ranges from 27% (India) [16] to 71%(Ghana) [17]. It could be concluded that most countries were not to reach the WHO global immunization target of at least 90% of the national TT vaccination coverage and at least 80% of coverage in every district [18, 19].

Age of the mothers, educational status, marital and occupational status, joint health decision with husband, distance from health facilities, wealth index, fear of side effects, fear of sterility, ANC follow-up, use of modern family planning, parity, lack of information about TT vaccination, knowledge, attitude, and low awareness of mothers were factors for TT^2+^ immunization coverage [14, 16, 18, 20–23]. TT^2+^ immunization status also differs from urban to rural mothers, as well as region to region in different countries [24, 25].

Despite the country's effort in interventional policy to meet the WHO goals for maternal and neonatal tetanus elimination (MNTE), Ethiopia continues to have the highest neonatal tetanus-related mortality and morbidity. The reasons are due to the low coverage of TT immunization and the high number of home deliveries in the country [26, 27]. Although the country was expected to reach 86% coverage of national tetanus protective at birth (PAB) by 2015, there is still low coverage and varies between regions with fewer identified associated factors [27, 28].

Studies in Ethiopia showed that the coverage of receiving at least two doses of tetanus toxoid immunization ranges from 13.9% (South region) to 79.0% (Amhara region) [29, 30]. This indicates high variation of tetanus toxoid coverage among geographical regions in the country. Furthermore, there is no nationally represented pooled data on tetanus toxoid immunization coverage in Ethiopia. Thus, reliable and summarized information is essential to refine government policies, strategies, and interventions. Therefore, the main objective of this systematic review and meta-analysis was to estimate the pooled coverage of at least two doses of tetanus toxoid immunization and associated factors in Ethiopia. This review can be of vital importance in showing summarized evidence and suggesting possible applicable strategies for planning, decision making, and resource allocation in the Ethiopian health care system.

## 2. Review Question

The review questions for this systematic review and meta-analysis were as follows:

- What is the pooled coverage of receiving at least two doses of tetanus toxoid immunization among childbearing women in Ethiopia from 2010 to 2020?
- What is the pooled effect size of factors associated with receiving at least two doses of tetanus toxoid immunization among childbearing women in Ethiopia from 2010 to 2020?

## 3. Methods

### 3.1. Information Source

The systematic review and meta-analysis were carried out following the guidelines of the Preferred Reporting Items for Systematic Reviews and Meta-Analyses (PRISMA) guidelines [31] (supplementary file 1). Published and unpublished (grey literature) research reports describing the coverage of tetanus toxoid immunization and its associated factors in Ethiopia were reviewed.

### 3.2. Eligibility Criteria

Cross-sectional and case-control studies with original data reporting the coverage of TT^2+^ immunization and/or its associated factors among childbearing women in Ethiopia published from 2010 to 2020 were included (The authors were interested to show the coverage of TT^2+^ for 10 years since maternal health service utilization assumed to be improved in the last 10 years according to the government reports). In this review, studies published in the English language or have English language versions were included. Studies that did not report specific outcomes for receiving at least two doses of tetanus toxoid immunization were excluded.

### 3.3. Outcomes of Measurement

This study has two main outcomes. The first outcome was the pooled coverage of TT^2+^ immunization in Ethiopia. The second outcome was pooled effect sizes of associated factors with TT^2+^ immunization. The coverage of TT^2+^ vaccination was calculated by dividing the number of mothers who have taken at least two doses of TT by the total number of mothers who were included in the study and multiplied by one hundred (100). For the second objective, the odds ratio was used to measure the level of the association between TT^2+^ immunization coverage and its associated factors. The odds ratio was calculated from primary studies using two-by-two tables.

### 3.4. Search Strategies

Relevant studies were searched from the PubMed/MEDLINE online, ScienceDirect, and Hinari databases. Grey literature was also identified from Google and Google Scholars. The last search date was 30 August 2020. We used the following search terms to search studies from databases: coverage/utilization/protective dose of tetanus toxoid immunization, tetanus toxoid immunization status, uptake of tetanus toxoid vaccination, and protection of the last live birth against neonatal tetanus. The key terms used to retrieve primary studies were (Utilization OR coverage OR vaccination uptake OR immunization status AND (Tetanus toxoid) AND Ethiopia). We also used key terms of (Factors OR determinants OR risk factors OR correlates) AND (Tetanus toxoid) AND Ethiopia to search the literature on factors associated with the coverage of tetanus toxoid immunization. Primary studies were searched by JN and BG.

### 3.5. Study Selection and Quality Appraisal

Original study from Ethiopian settings was included in this review, whereas comments, editorials, and reviews were excluded. The primary investigator performed an initial review of the eligibility of the searched literature by reading their titles and abstracts. The full-text articles were included if they reported the coverage of TT^2+^ immunization and/or its associated factors. Two reviewers (BG and AM) independently screened the selected full-text articles using prespecified inclusion criteria using a uniform assessment tool. During the selection process, disagreements between the two authors were resolved by mediation of the fourth reviewer (MM) to include the final decision in the analysis.

The qualities of the primary studies were assessed using the Newcastle-Ottawa scale [32]. The tool has three main parts. The first part has five components used to assess the methodological quality of each study. The second part assesses the comparability of primary studies, and the final part of the tool measures the quality of the original articles with respect to their outcome and statistical analysis (supplementary file 2). Two authors (JN and BG) independently evaluated the qualities of each original article. Any difference between the two authors during the quality assessment was solved by taking the average of the two assessment scores. All articles scored 6 and more can be considered as low risk and good to be included for the meta-analysis.

### 3.6. Data Extraction

We used a standardized data extraction format prepared in Microsoft Excel to extract all the necessary data. The extraction format prepared in different columns contains the name of the first author, publication year, the region where the study was conducted, sample size, response rate, and coverage of TT^2+^ immunization for the first objective. For the second objective (factors associated with TT^2+^ immunization), the data extraction format was prepared in the form of a two-by-two table. Antenatal care, distance from the health facility, media exposure, educational status of women, and education status of their husbands were extracted from each study. These categorical variables were tabulated (a, b, c, and d) with the outcome variable during extraction. Data were extracted by two authors (JN and BG) using a standardized data extraction format. The third and fourth authors (AM and MM) evaluated the accuracy of the extracted data.

### 3.7. Data Analysis and Interpretation

The data from the Microsoft Excel were exported to STATA Version 14.0 (software) for analysis. A random-effects meta-analysis model was used, since it reduces heterogeneity among studies. The pooled effect size was conducted in the form of prevalence and odds ratio. Statistical heterogeneity was evaluated by the *I*^2^ test, which shows the level of heterogeneity between studies [33]. Furthermore, we also employed subgroup and leave-one-out sensitivity analysis to identify the possible source of heterogeneity in the pooled meta-analysis. Egger's weighted regression test was used to assess publication bias at a 5% significance level [34, 35]. *P* < 0.05 was considered statistically significant publication bias.

## 4. Results

### 4.1. Search Results

**Figure 1:**
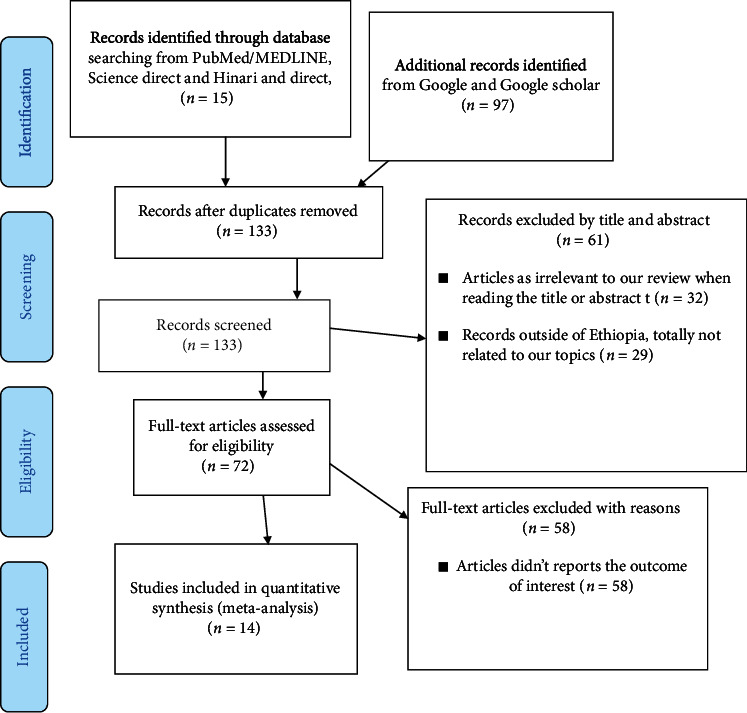
PRISMA 2020 flow diagram for systematic reviews which included searches of databases and registers only.

There were a total of 212 primary records retrieved during the literature search from the PubMed/MEDLINE online, ScienceDirect, Hinari, Google, and Google Scholar databases. About 79 records were excluded due to duplication, and 61 articles were excluded after reading their title and abstract. After the full-text review of the remaining articles, 58 articles were further excluded with reason. Finally, 14 primary studies were included in this meta-analysis (Figure 1).

### 4.2. Characteristics of the Included Articles

In this review, 14 primary studies with a total of 14,429 study participants were included. The studies published from 2010 to 2020 were retrieved from five regions of the country (four studies were conducted in the regional state of the South Nations Nationalities and Peoples of Ethiopia, three in the regional state of Amhara, four in the regional state of Oromia, one in the regional state of Tigray, one in the regional state of Somalia, and one study was national wide based on EDHS data [29, 30, 36–47]). All these studies included in this review were community-based cross-sectional studies with sample sizes ranging from 239 to 7,193 participants as reported from studies done in Bahir Dar and from EDHS data, respectively [36, 41]. Therefore, each study used simple or systematic random samplings to specify that the risk of selection bias was not a significant problem. Six studies were conducted in the urban area, and seven studies were conducted in the rural area. The highest coverage of TT^2+^ immunization was reported from a study conducted in Debre Tabor town in the Amhara region (79.0%) [41], and the lowest was from a study conducted in the Meinit-Shasha district in the southern region (13.9%) [29] (Table 1).

### 4.3. Tetanus Toxoid Vaccination Coverage

**Figure 2:**
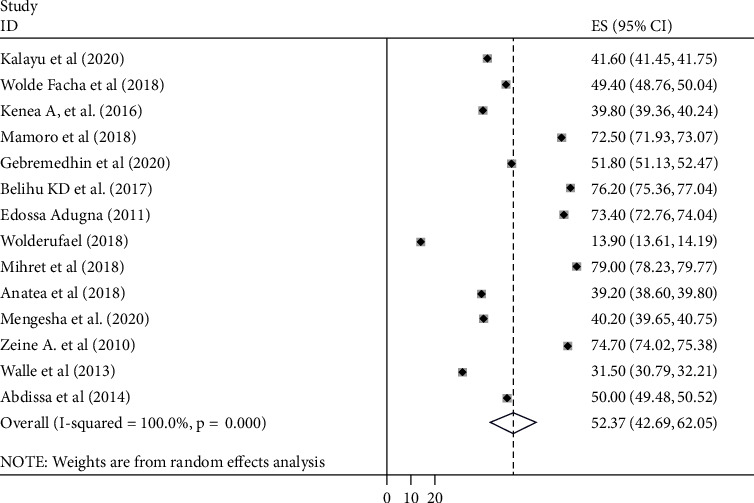
Forest plot for the pooled coverage of tetanus toxoid immunization in Ethiopia, 2020.

**Figure 3:**
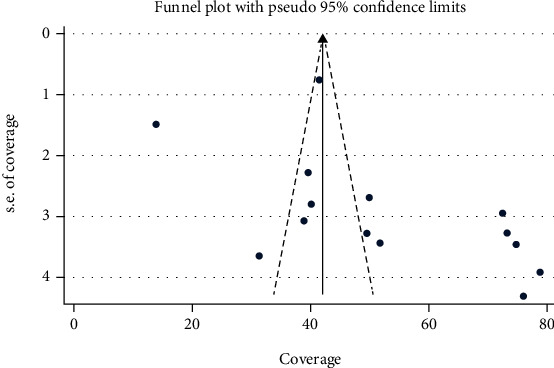
Funnel plot showing the symmetric distribution of articles of tetanus toxoid immunization in Ethiopia, 2020.

A random-effects model was used to estimate the pooled coverage of TT^2+^ immunization among childbearing women in Ethiopia. The pooled estimate of at least two doses of tetanus immunization coverage in Ethiopia was 52.4% (95% CI: 42.69-62.03, *I*^2^ = 98.4%) (Figure 2). Publication bias was checked using Egger's test (*P* = 0.080), which showed that there was no significant publication bias. We also observed a symmetrical distribution of the funnel plot indicating that publication bias is not a significant problem (Figure 3). Furthermore, we also conducted a leave-one-out sensitivity analysis to identify the possible source of heterogeneity in the pooled meta-analysis. The test results showed that the pooled coverage of TT^2+^ immunization ranges between 50.4% (39.2–61.5, *I*^2^ = 97.4%) and 54.4% (42.6–66.0, *I*^2^ = 98.3%). This indicates that the result of the review was strong and did not depend on the addition or removal of a single study from the analysis (Table 2).

Subgroup analysis was also performed using region, sampling technique, study area, and year of publication (Table 3).

#### 4.3.1. Subgroup Analysis by Region

The pooled TT^2+^ immunization coverage in the Amhara region was 62.3% ((95% CI: 34.1, 67.2), *I*^2^ = 99.0%) and 50.6% in the Oromia region ((95% CI: 27.3, 74.3), *I*^2^ = 89.2).

#### 4.3.2. Subgroup Analysis Using Sampling Technique

The pooled coverage of two or more tetanus toxoid immunization in multistage, simple random, and systematic random sampling technique was 57.5% ((95% CI: 43.7, 71.0), *I*^2^ = 96.5%), 39.3% ((95% CI: 12.1, 66.3), *I*^2^ = 99.1%), and 61.8% ((95% CI: 42.6, 80.6), *I*^2^ = 96.1%), respectively.

#### 4.3.3. Subgroup Analysis by Study Area

This meta-analysis also revealed that the pooled coverage of two or more TT immunizations was slightly higher in urban women (56.46%, (95% CI: 39.7-73.28), *I*^2^ = 97.3%) than in rural women (50.2% (95% CI: 32.75-68.67), *I*^2^ = 98.9%).

#### 4.3.4. Subgroup Analysis by Publication Year

The pooled coverage of TT^2+^ immunization among childbearing women in Ethiopia was found to be 57.41% (95% CI: 38.47-76.34, *I*^2^ = 97.2%) from studies published from January 2010 to December 2015, while it was 50.12% (95% CI: 38.89–61.34, *I*^2^ = 98.6%) from studies published from January 2016 to August 2020.

### 4.4. Factors Associated with Coverage of Tetanus Toxoid Immunization

Eight primary studies were included in this meta-analysis to estimate the size of the pooled effect size of factors associated with receiving two or more coverage of tetanus toxoid immunizations in Ethiopia. Variables reported as factors in at least three studies were included in the metal analysis. Random-effects meta-analysis models that consider heterogeneity among studies were used to identify factors significantly associated with tetanus toxoid immunization coverage. Accordingly, antenatal care visit [6], media exposure, distance of the health facility, and educational status of mothers and their husbands were found to have a significant association with coverage of tetanus toxoid immunization in Ethiopia.

Antenatal care follow-up during pregnancy was identified as a factor for tetanus toxoid immunization coverage among six primary studies included in this meta-analysis [30, 36, 40, 42–44]. A total of 9,755 study participants were included to analyze the association between ANC visits and tetanus toxoid immunization. Mothers who had ANC visits during the last pregnancy had 7.8 times more chances of receiving two or more doses of tetanus toxoid immunization compared to their counterparts (OR = 7.8 (95% CI: 3.2, 19.2), *I*^2^ = 96.3%) (Table 4, supplementary file three).

Three primary studies included in our meta-analysis reported that exposure to the media (TV and radio) was significantly associated with TT^2+^ immunization coverage [36, 42, 43]. To see the association between media exposure and tetanus toxoid vaccination coverage, 8,289 study participants were included in the analysis. Accordingly, tetanus toxoid immunization coverage among women who had media exposure was 8.3 times higher than women who had no media exposure (OR = 8.3 (95% CI: 2.1, 33.3), *I*^2^ = 98.1%)) (Table 4, supplementary file three).

The distance from home to the health facility was identified as a factor associated with receiving two or more doses of tetanus toxoid immunization among three primary studies with a total of 1,933 study participants [40, 43, 44]. Women who walk less than 1 hour to reach the health facility had 2.6 times higher to receive at least two doses of tetanus toxoid immunization than women who walk more than or equal to 1 hour to reach the health facility (OR = 2.64 (95% CI: 1.1, 6.6), *I*^2^ = 94.1%) (Table 4, supplementary file three).

The educational status of the mothers was significantly associated with the coverage of tetanus toxoid immunization among five primary studies included in our analysis [30, 39, 40, 42, 43]. A total of 2,884 women were included in the analysis to examine the association between the educational status of mothers and tetanus toxoid immunization coverage. Educated women had 4.5 times higher to receive two or more doses of TT injection than noneducated women (OR = 4.7 (95% CI: 2.07, 9.56), *I*^2^ = 94.2%) (Table 4, supplementary file three).

Three primary studies reported that the husband's educational status was associated with receiving two or more doses of tetanus toxoid immunization with a total sample of 1,957 women [39, 40, 42]. Accordingly, women who have educated husbands are 3 times higher to receive two or more tetanus toxoid immunizations compared to women whose husbands are not educated (OR = 2.95 (95% CI; 1.194, 7.512), *I*^2^ = 92.5%) (Table 4, supplementary file three).

## 5. Discussion

Neonatal and maternal tetanus is still a major public health problem, especially in developing countries, which can be easily prevented by immunization of childbearing women with at least two doses of the tetanus toxoid immunization. This systematic review and meta-analysis was conducted to show the pooled coverage of two or more doses of tetanus toxoid immunization and associated factors in Ethiopia. This is the first systematic review and meta-analysis on the topic of tetanus toxoid immunization in the country.

In this meta-analysis, the pooled coverage of at least two doses of tetanus toxoid immunization was 52.4% (95% CI: 42.69-61.03, *I*^2^ = 98.4%). This finding was consistent with a report of studies conducted in Kenya (52.0%) and Pakistan (55.6%) [18, 48]. It was low compared to studies conducted in other developing countries such as Ghana 71% [49], India 68% [16], and Sierra Leone 82.1% [50]. The possible explanation for this difference could be due to geographical differences, sociocultural variation, and maternal health service utilization between the countries. The variation might also be due to the nature of the studies between meta-analysis and primary studies. However, the pooled coverage of at least two doses of tetanus toxoid immunization in this meta-analysis was higher as compared to studies conducted in Rivers State, Nigeria 37.1% [51] and Nigeria 40.8% [52]. The discrepancy could be due to the difference in the number of study participants between studies.

The second objective of this study was to identify factors associated with receiving at least two doses of tetanus toxoid immunization among childbearing women. Accordingly, ANC visits, media exposure, distance of health facilities, and educational status of mothers and their husbands were significantly associated with coverage of tetanus toxoid immunization among women of childbearing age in Ethiopia. The coverage of tetanus toxoid immunization was 7.8 times higher among mothers who attended ANC follow-up compared to their counterparts. This finding was supported by studies conducted in Kenya [21] and Pakistan [18]. Tetanus toxoid immunization was one of the routine interventions during ANC visits, and counselling about the importance of TT immunization can increase maternal awareness on the advantage of continuous immunization.

Women who had media exposure were 8.3 times more likely to receive two or more doses of tetanus toxoid immunization than women who had no media exposure. A similar finding was reported from a study conducted in Indonesia [53]. The possible explanation for the association of media exposure with tetanus toxoid immunization might be that the media may provide important information that can increase women's knowledge on the advantage of tetanus toxoid immunization. The chance of receiving two or more doses of tetanus toxoid immunization was 2.6 times higher among women who walk less than 1 hour to reach the nearest health facility than among women who walk more than 1 hour to reach the nearest health facility. This could be because women in the home are responsible for multiple tasks that make them busy to receive tetanus toxoid immunization, since TT vaccination requires repeated visits to health facilities.

Educated women in this meta-analysis were 4.4 times more likely to receive two or more TT immunizations than uneducated women. This is consistent with studies conducted in Bangladesh [54] and France [55]. This could be because education increases women's knowledge, attitude, and awareness on the health benefit of tetanus toxoid immunization. Similarly, women with an educated husband were 3 times higher to receiving two or more tetanus toxoid immunizations than women with uneducated husbands. This could be because educated husbands have better knowledge and awareness of the benefits of TT immunization that could drive their wives to take the immunization. Furthermore, educated husbands may give more freedom to their wives to receive tetanus toxoid vaccination compared to noneducated husbands. The current systematic review and meta-analysis showed that the coverage of tetanus toxoid immunization in Ethiopia (52.4%) is very low and far from WHO's recommendation at the national level (90%) [19]. This study presents companied information regarding the coverage of tetanus toxoid immunization and associated factors. Therefore, it was alarming stakeholders to act cooperatively to improve TT immunization coverage in order to reduce maternal and neonatal tetanus.

### 5.1. Limitation of the Study

Most of the primary studies included in this systematic review and meta-analysis were cross-sectional studies that are difficult to establish cause-effect relationships. The presence of significant heterogeneity between the primary studies is the other limitation of this study. Limited to articles published in English language is also considered as a limitation for this study.

## 6. Conclusion

This systematic review and meta-analysis showed that the coverage of tetanus toxoid immunization among childbearing women in Ethiopia was low compared to the WHO global immunization target. It was higher in urban women than in rural women. The absence of ANC visits, no media exposure, distant health facilities, and low educational status were factors for the low coverage for tetanus toxoid immunization. Strengthening maternal health service utilization, such as ANC, to the nearest health facility, including in health posts is recommended to increase the coverage of tetanus toxoid immunization. Empowering education and media exposure (TV and radio) also plays an important role to increase tetanus toxoid immunization coverage.

## Figures and Tables

**Table 1:** Summary of the studies included in the systematic review and meta-analysis of tetanus toxoid immunization coverage and associated factors in Ethiopia 2020.

Author	Publication year	Region	Sample size	TT^2+^ coverage	Study design	Study area	Sampling technique	Reference
Kalayu et al.	2020		7193	41.6			Multilevel analysis	[36]
Facha et al.	2018	South	462	49.4	Cross-sectional	Rural	Multistage	[37]
Kenea et al.	2016	Oromia	779	39.8	Cross-sectional	Rural	Multistage	[38]
Mamoro et al.	2018	South	837	72.5	Cross-sectional	Rural	Simple random	[39]
Gebremedhin et al.	2020	Somalia	440	51.8	Cross-sectional	Rural	Systematic random	[40]
Belihu et al.	2017	Amhara	408	76.2	Cross-sectional	Urban	Systematic random	[41]
Adugna	2011	Oromia	680	73.4	Cross-sectional	Urban	Multistage	[42]
Wolderufael	2018	South	639	13.9	Cross-sectional	Rural	Simple random	[29]
Mihret et al.	2018	Amhara	511	79	Cross-sectional	Urban	Systematic random	[30]
Anatea et al.	2018	Oromia	416	39.2	Cross-sectional	Urban	Simple random	[43]
Mengesha et al.	2020	Tigray	515	40.2	Cross-sectional	Urban	Systematic random	[44]
Zeine et al.	2010	South	612	74.7	Cross-sectional	Rural	Multistage sampling	[46]
Walle et al.	2013	Amhara	239	31.5	Cross-sectional	Urban	Simple random	[45]
Abdissa et al.	2014	Oromia	698	50	Cross-sectional	Rural	Multistage	[47]

TT^2+^: two or more dose of tetanus toxoid immunization.

**Table 2:** Sensitivity analysis in the systematic review and meta-analysis of the coverage of tetanus toxoid immunization and associated factors in Ethiopia, 2020.

Authors	Publication year	Two or more tetanus toxoid immunization coverage	95% confidence interval	Reference
Kalayu et al.	2020	53.5	39.7-67.2	[36]
Facha et al.	2018	52.8	40.9-64.8	[37]
Kenea et al.	2016	53.7	41.5-65.7	[38]
Mamoro et al.	2018	50.9	39.5-62.4	[39]
Gebremedhin et al.	2020	52.6	40.7-64.6	[40]
Belihu et al.	2017	50.6	39.2-62.0	[41]
Adugna	2011	50.8	39.4-62.3	[42]
Wolderufael	2018	55.8	45.6-66.0	[29]
Mihret et al.	2018	50.4	39.2-61.5	[30]
Anatea et al.	2018	53.7	41.8-65.6	[43]
Mengesha et al.	2020	53.6	41.7-65.6	[44]
Zeine et al.	2010	50.7	39.3-62.1	[46]
Walle et al.	2013	54.3	42.6-66.0	[45]
Abdissa et al.	2014	52.384	41.9-62.8	[47]

**Table 3:** Summary of the subgroup analysis for the systematic review and meta-analysis of tetanus toxoid immunization coverage and associated factors in Ethiopia, 2020.

Type	Feature	Number of studies	Pooled coverage of TT^2+^ immunization, % (95% CI)	*I* ^2^ (*P* value)
Subgroup analysis by region	Amhara region	3	62.19 (30.74–93.62)	98.0% (<0.01)
	Oromia region	4	50.50 (36.28-64.74)	96.4% (<0.01)
	South region	4	52.55 (212.66-86.45)	99.4 (<0.01)
	Somalia region^∗^	1	51.80 (45.09-58.50)	—
	Tigray region^∗^	1	40.20 (34.73–45.66)	—

Subgroup analysis by sampling technique	Multistage sampling	5	57.33 (43.65-71.01)	96.5 (<0.01)
	Simple random sampling	4	39.28 (12.11–66.34)	99.1 (<0.01)
	Systematic random sampling	4	61.63 (42.64-80.62)	96.1 (<0.01)

Subgroup analysis by study area	Rural	7	50.21 (32.75-68.67)	98.9 (<0.01)
	Urban	6	56.46 (39.70–73.28)	97.3 (<0.01)

Subgroup analysis by publication year	January 2010-December 2015	4	57.41 (38.47-76.34)	97.2 (<0.01)
	January 2016-August 2020	10	50.12 (38.89-61.34)	98.6 (<0.01)

^∗^Regions having a single study. TT^2+^: two or more doses of tetanus toxoid immunization.

**Table 4:** Summary of factors associated with tetanus toxoid immunization coverage in Ethiopia, 2020.

Variables	Number of studies	Odds ratio with 95% CI	Heterogeneity	
			*I* ^2^	*P* value
Antinatal care	6	7.8 (3.2, 19.2)	96.3%	*P* < 0.01
Media exposure	3	8.3 (2.1, 33.3)	98.1%	*P* < 0.01
Distance of health facility	3	2.64 (1.1, 6.6)	94.1%	*P* < 0.01
Women's educational status	5	4.7 (2.07, 9.56)	94.2%	*P* < 0.01
Husband educational status	3	2.95 (1.2, 7.51)	92.5%	*P* < 0.01

## Data Availability

The data used for this study are available here. It will be shared upon request and will be obtained by email to the corresponding author using “jemberu2123@gmail.com.”

## Abbreviations

ANC: Antenatal care
TT: Tetanus toxoid
TT^2+^: Two or more doses of tetanus toxoid immunization
WHO: World Health Organization
SEA: South East Asian
SSA: Sub-Saharan African
MNT: Maternal and neonatal tetanus
CBAW: Women of childbearing age
MNTE: Maternal and neonatal tetanus elimination
PAB: Protective at birth
EDHS: Ethiopia demographic health survey
PRISMA: Preferred reporting items for systematic reviews and meta-analysis.

## Supplementary Materials

Supplementary Materials We have uploaded two important files as supplementary material. The first file is the PRISMA check list that was used as a guild line to carry out systematic review and meta-analysis. This systematic review and meta-analysis was performed following the PRISMA guidelines. The second file is a quality score table that shows the quality score of the primary studies included in this systematic review and meta-analysis. The quality score for each study was assessed using the Newcastle-Ottawa assessment scale.
