# Supplementary material for: Tetanus Toxoid Vaccination Coverage and Associated Factors among Childbearing Women in Ethiopia: A Systematic Review and Meta-Analysis
Source: Biomed Res Int. 2021 Nov 8;2021:5529315. doi: 10.1155/2021/5529315 (PMC8592723; doi:10.1155/2021/5529315)
Supplement: Supplementary Materials — We have uploaded two important files as supplementary material. The first file is the PRISMA check list that was used as a guild line to carry out systematic review and meta-analysis. This systematic review and meta-analysis was performed following the PRISMA guidelines. The second file is a quality score table that shows the quality score of the primary studies included in this systematic review and meta-analysis. The quality score for each study was assessed using the Newcastle-Ottawa assessment scale. [file 5529315.f1.zip › Quaity score (suplmentry two) docx.docx]

**Table 1:** Quality score based on Newcastle-Ottawa Scale of studies included in the systematic review and meta-analysis of tetanus toxoid immunization coverage and associated factors in Ethiopia based on 2020.

| Author | Publication year | Sample size | Response rate | TT^2+^ coverage | Quality score | | | |
| --- | --- | --- | --- | --- | --- | --- | --- | --- |
|  |  |  |  |  | Selection (from 5 point) | Comparability (from 2 point) | Outcome (from 3 point) | Overall score (from 10 point) |
| Kalayu et al | 2020 | 7193 | 100 | 41.6 | 4 | 1 | 3 | 8 |
| Wolde Facha et al | 2018 | 462 | 100 | 49.4 | 3 | 2 | 2 | 7 |
| Kenea A, et al. | 2016 | 779 | 100 | 39.8 | 4 | 2 | 2 | 8 |
| Mamoro et al | 2018 | 837 | 99 | 72.5 | 4 | 2 | 3 | 9 |
| Gebremedhin et al | 2020 | 440 | 98 | 51.8 | 4 | 2 | 3 | 9 |
| Belihu KD et al. | 2017 | 408 | 96.6 | 76.2 | 3 | 1 | 2 | 6 |
| Edossa Adugna | 2011 | 680 | 100 | 73.4 | 4 | 1 | 2 | 7 |
| Wolderufael | 2018 | 639 | 98.1 | 13.9 | 4 | 1 | 2 | 7 |
| Mihret et al | 2018 | 511 | 100 | 79 | 3 | 2 | 2 | 7 |
| Anatea et al | 2018 | 416 | 98.6 | 39.2 | 4 | 2 | 3 | 9 |
| Mengesha et al. | 2020 | 515 | 100 | 40.2 | 4 | 2 | 2 | 8 |
| Zeine A. et al | 2010 | 612 | 86.2 | 74.7 | 3 | 1 | 2 | 6 |
| Walle et al | 2013 | 239 | 100 | 31.5 | 2 | 2 | 2 | 6 |
| Abdissa et al | 2014 | 698 | 95 | 50 | 3 | 3 | 3 | 9 |
