# Supplementary material for: Tetanus Toxoid Vaccination Coverage and Associated Factors among Childbearing Women in Ethiopia: A Systematic Review and Meta-Analysis
Source: Biomed Res Int. 2021 Nov 8;2021:5529315. doi: 10.1155/2021/5529315 (PMC8592723; doi:10.1155/2021/5529315)
Supplement: Supplementary Materials — We have uploaded two important files as supplementary material. The first file is the PRISMA check list that was used as a guild line to carry out systematic review and meta-analysis. This systematic review and meta-analysis was performed following the PRISMA guidelines. The second file is a quality score table that shows the quality score of the primary studies included in this systematic review and meta-analysis. The quality score for each study was assessed using the Newcastle-Ottawa assessment scale. [file 5529315.f1.zip › supplmentry file three .docx]

**Figure 4:** Association of anti-natal care visits with coverage of tetanus toxoid immunization in Ethiopia, 2020.

**Figure 5:** Association of media exposure with tetanus toxoid immunization coverage in Ethiopia, 2020.

**Figure 6:** Association of the distance from the health facility with the coverage of tetanus toxoid immunization in Ethiopia, 2020

**Figure 7:** Association of women’s educational status with coverage of tetanus toxoid immunization in Ethiopia, 2020.

NOTE: Weights are from random effects analysis

Overall (I-squared = 94.2%, p < 0.01)

Gebremedhin et al (2020)

Anatea et al (2018)

Study

EdossaAdugna (2011)

Mamoro et al (2018)

Mihret et al (2018)

ID

4.45 (2.07, 9.56)

16.30 (9.95, 26.69)

3.08 (2.03, 4.67)

1.83 (1.28, 2.62)

7.95 (5.48, 11.55)

2.45 (1.56, 3.85)

OR (95% CI)

4.45 (2.07, 9.56)

16.30 (9.95, 26.69)

3.08 (2.03, 4.67)

1.83 (1.28, 2.62)

7.95 (5.48, 11.55)

2.45 (1.56, 3.85)

OR (95% CI)

1

.1

1

10

NOTE: Weights are from random effects analysis

Overall (I-squared = 92.5%, p < 0.01)

Gebremedhin et al (2020)

Study

EdossaAdugna (2011)

Mamoro et al (2018)

ID

3.00 (1.19, 7.51)

1.21 (0.79, 1.85)

6.99 (4.04, 12.09)

3.29 (2.30, 4.72)

OR (95% CI)

3.00 (1.19, 7.51)

1.21 (0.79, 1.85)

6.99 (4.04, 12.09)

3.29 (2.30, 4.72)

OR (95% CI)

1

.1

1

10

**Figure 8:** Association of the educational status of the husband with maternal tetanus toxoid immunization coverage in Ethiopia, 2020.
